# Similar rate of venous thromboembolism (VTE) and failure of non-operative management for early versus delayed VTE chemoprophylaxis in adolescent blunt solid organ injuries: a propensity-matched analysis

**DOI:** 10.1007/s00068-023-02440-4

**Published:** 2024-01-09

**Authors:** Areg Grigorian, Sebastian Schubl, Lourdes Swentek, Cristobal Barrios, Michael Lekawa, Dylan Russell, Jeffry Nahmias

**Affiliations:** 1 Division of Trauma, Burns and Surgical Critical Care, Department of Surgery, University of California, Irvine Medical Center, 3800 Chapman Ave, Suite 6200, Orange, CA 92868-3298 USA; 2 Department of Surgery, University of Southern California, Los Angeles, CA USA

**Keywords:** Pediatric trauma, Blunt solid organ injury, Venous thromboembolism chemoprophylaxis, Deep vein thrombosis, Pulmonary embolism

## Abstract

**Background:**

Early initiation of venous thromboembolism (VTE) chemoprophylaxis in adults with blunt solid organ injury (BSOI) has been demonstrated to be safe but this is controversial in adolescents. We hypothesized that adolescent patients with BSOI undergoing non-operative management (NOM) and receiving early VTE chemoprophylaxis (eVTEP) (≤ 48 h) have a decreased rate of VTE and similar rate of failure of NOM, compared to similarly matched adolescents receiving delayed VTE chemoprophylaxis (dVTEP) (> 48 h).

**Methods:**

The 2017–2019 Trauma Quality Improvement Program database was queried for adolescents (12–17 years of age) with BSOI (liver, kidney, and/or spleen) undergoing NOM. We compared eVTEP versus dVTEP using a 1:1 propensity score model, matching for age, comorbidities, BSOI grade, injury severity score, hypotension on arrival, and need for transfusions. We performed subset analyses in patients with isolated spleen, kidney, and liver injury.

**Results:**

From 1022 cases, 417 (40.8%) adolescents received eVTEP. After matching, there was no difference in matched variables (all *p* > 0.05). Both groups had a similar rate of VTE (dVTEP 0.6% vs. eVTEP 1.7%, *p* = 0.16), mortality (dVTEP 0.3% vs. eVTEP 0%, *p* = 0.32), and failure of NOM (eVTEP 6.7% vs. dVTEP 7.3%, *p* = 0.77). These findings remained true in all subset analyses of isolated solid organ injury (all *p* > 0.05).

**Conclusions:**

The rate of VTE with adolescent BSOI is exceedingly rare. Early VTE chemoprophylaxis in adolescent BSOI does not increase the rate of failing NOM. However, unlike adult trauma patients, adolescent patients with BSOI receiving eVTEP had a similar rate of VTE and death, compared to adolescents receiving dVTEP.

**Supplementary Information:**

The online version contains supplementary material available at 10.1007/s00068-023-02440-4.

## Background

In the USA, trauma remains the foremost cause of mortality among adolescents [1]. Approximately 15% of injured children exhibit intra-abdominal injuries, with the spleen being the most frequently affected organ [2]. For hemodynamically stable pediatric patients with blunt solid organ injury (BSOI), non-operative management (NOM) is now the standard of care, exhibiting a relatively low failure rate of 5–10%, even for high-grade injuries such as American Association for the Surgery of Trauma grades III–V [3, 4]. The primary cause of NOM failure is bleeding, which may be aggravated by the introduction of venous thromboembolism (VTE) chemoprophylaxis.

Pediatric and adolescent trauma patients display a VTE rate of less than 2%, a stark contrast to the adult VTE rate that can reach up to 60% [5–7]. In adult BSOI cases, prospective studies have shown that early VTE chemoprophylaxis initiation can mitigate VTE risk without increasing NOM failure [8, 9]. Optimal timing for early chemoprophylaxis appears to be within 48 h post-injury, provided there are no active bleeding signs, as this threshold marks a potential transition from a hypocoagulable to a hypercoagulable state [10]. However, considerable variation exists among trauma centers in terms of VTE chemoprophylaxis initiation across diverse populations, including adult BSOI patients [11, 12].

Several key distinctions exist between adult and pediatric BSOI patients. In pediatric cases, factors such as serial vital signs, BSOI grade, and intraperitoneal fluid on imaging do not correlate with outcomes [13]. Moreover, children may lose nearly 40% of their circulating blood volume before displaying hypotension, complicating early bleeding detection. Consequently, trauma surgeons may exercise greater caution in initiating early VTE chemoprophylaxis for pediatric BSOI patients [14].

Given the exceedingly rare VTE rate in children under 12, most providers do not administer VTE chemoprophylaxis to trauma patients in this age group [15]. The majority of VTE events in hospitalized children occur in adolescents (12–17 years old), prompting trauma surgeons to consider VTE chemoprophylaxis in this demographic [16–19]. However, there is a scarcity of literature to inform decision-making on VTE chemoprophylaxis initiation in adolescents with BSOI, especially when utilizing a national sample. Therefore, we hypothesized that adolescents with BSOI undergoing NOM and receiving early VTE chemoprophylaxis (eVTEP) (within 48 h) would experience a decreased VTE rate and a similar NOM failure rate compared to similarly matched adolescents receiving delayed VTE chemoprophylaxis (dVTEP) (beyond 48 h).

## Methods

This study was exempt from approval by our institutional review board, and thus, no consent needed, as it utilizes a national deidentified database. We report our findings in accordance with the Strengthening the Reporting of Observational Studies in Epidemiology (STROBE) statement (Supplemental File 1). We queried the 2017–2019 Trauma Quality Improvement Program (TQIP) database for adolescents aged 12–17 years with BSOI (kidney, liver, or spleen) who underwent non-operative management (NOM).

Exclusion criteria were traumatic brain injury, pre-existing anticoagulation/coagulopathy, exploratory laparotomy within 2 h of arrival, inter-hospital transfer, and lack of VTE chemoprophylaxis during hospitalization. Patients who died or were discharged within 48 h of arrival were also excluded. The early discharge group was excluded for several reasons. Oftentimes, these patients are not severely injured and may have contraindications for VTE chemoprophylaxis including being sufficiently ambulatory. Additionally, we did not want to include patients that may have had VTE on admission as our intent was to design a study to analyze the impact of VTE chemoprophylaxis in patients without VTE on admission. Patients receiving eVTEP (≤ 48 h of arrival) were compared to those receiving dVTEP (> 48 h of arrival). To compare similar patient groups, we employed a 1:1 propensity score model matching for age, comorbidities, angiography use, solid organ injury, BSOI grade (defined by the abbreviated injury scale grade for the abdomen), pelvic fracture, long bone fracture, injury severity score (ISS), hypotension on arrival (systolic blood pressure < 90 mmHg), and blood transfusion necessity. Matched comorbidities included hypertension, attention-deficit hyperactivity disorder, psychiatric illness, smoking, substance abuse, and diabetes. Propensity matching was performed using cases within 0.001 of the estimated logit [20].

Our primary outcome was the composite VTE rate, comprising deep vein thrombosis (DVT) and pulmonary embolism (PE). The secondary outcome was the rate of NOM failure. Additional outcomes included total hospital length of stay (LOS), intensive care unit (ICU) LOS, and mortality rate. Bivariate analyses were conducted using the Mann–Whitney *U* test for continuous variables and the chi-square test for categorical variables. Categorical data was reported as percentages, while continuous data was presented as medians with interquartile range or means with standard deviation.

We performed several additional subset analyses. First, we selected for isolated abdominal injuries, excluding patients with AIS grades > 2 for the head, spine, thorax, and upper and lower extremities. Next, we analyzed three distinct groups: isolated splenic injuries (no concurrent liver or kidney injuries), isolated liver injuries (no concurrent spleen or kidney injuries), and isolated kidney injuries (no concurrent spleen or liver injuries). For each group, we performed bivariate analyses to determine VTE and NOM failure rates. Failure of NOM was separately defined for each group. For splenic injuries, this included splenectomy or exploratory laparotomy performed > 48 h from admission. For liver injuries, this included control/repair of liver hemorrhage or exploratory laparotomy performed > 48 h from admission. For kidney injuries, this included nephrectomy or exploratory laparotomy performed > 48 h from admission. We additionally analyzed time to chemoprophylaxis to determine a relationship between time to chemoprophylaxis and VTE. The area under receiver operating characteristic (AUROC) curve was examined to evaluate this relationship. All *p*-values were two-sided, with a statistical significance level of < 0.05. IBM SPSS Statistics for Windows (Version 28, IBM Corp., Armonk, NY) was used for all analyses.

## Results

### Demographics of patients undergoing eVTEP and dVTEP initiation

A total of 1022 patients met the inclusion criteria, with 417 (40.8%) receiving eVTEP and 605 (59.2%) receiving dVTEP (Table 1). After matching, 358 eVTEP patients were compared to 358 dVTEP patients. After matching, we compared 358 eVTEP patients to 358 dVTEP patients, observing no differences in all matched variables between the groups (all *p* > 0.05). The most prevalent comorbidity among all patients was smoking (8.2%), and the spleen was the most commonly injured organ (54.3%). More than half of the patients (52.7%) had a high-grade solid organ injury (AIS grade > 3) (Table 2).

**Table 1:** Demographics for unmatched cohort of patients receiving early vs. delayed VTE chemoprophylaxis

Characteristic	eVTEP (*n* = 417)	dVTEP (*n* = 605)	*p*-value
Age, year, median (IQR)	16 (1)	16 (2)	0.043
Male, *n* (%)	251 (60.2%)	335 (55.4%)	0.126
Comorbidities, *n* (%)			
Hypertension	2 (0.5%)	1 (0.2%)	0.361
ADHD	22 (5.3%)	39 (6.4%)	0.438
Psychiatric illness	16 (3.8%)	39 (6.4%)	0.069
Smoker	41 (9.8%)	37 (6.1%)	0.028
Substance abuse	25 (6.0%)	26 (4.3%)	0.223
Diabetes	1 (0.1%)	3 (0.8%)	0.381
Angiography, *n* (%)	19 (4.6%)	50 (8.3%)	0.020
Hypotensive on admission, *n* (%)	17 (4.2%)	42 (7.0%)	0.060
Received PRBC transfusion within 4 h, *n* (%)	62 (15.9%)	151 (25.0%)	< 0.001
ISS, median (IQR)	17 (14)	22 (12)	< 0.001
Injury, *n* (%)			
Kidney	115 (27.6%)	193 (31.9%)	0.139
Spleen	217 (52.0%)	316 (52.2%)	0.952
Liver	200 (48.0%)	328 (54.2%)	0.049
Pelvic fracture	118 (28.3%)	201 (33.2%)	0.095
Long bone fracture (humerus, femur, tibia/fibula)	133 (31.9%)	243 (40.2%)	0.007
Severe spinal injury*	4 (1.0%)	18 (3.0%)	0.029
AIS-Abdomen grade, *n* (%)			< 0.001
2	217 (52.0%)	207 (34.2%)	
3	111 (26.6%)	189 (31.2%)	
4	66 (15.8%)	146 (24.1%)	
5	23 (5.5%)	63 (10.4%)	

*VTE* venous thromboembolism, *eVTEP* early VTE chemoprophylaxis, *dVTEP* delayed VTE chemoprophylaxis, *ADHD* attention-deficit hyperactivity disorder, *PRBC* packed red blood cells, *ISS* injury severity score, *AIS* abbreviated injury scale

^*^Defined by AIS grade ≥ 3 for the spine

**Table 2:** Demographics for 1:1 propensity score-matched patients receiving early vs. delayed VTE chemoprophylaxis

Characteristic	eVTEP (*n* = 358)	dVTEP (*n* = 358)	*p*-value
Age, year, median (IQR)	16 (1)	16 (1)	1.000
Male, *n* (%)	201 (56.1%)	215 (60.1%)	0.289
Comorbidities, *n* (%)			
Hypertension	1 (0.3%)	1 (0.3%)	1.000
ADHD	15 (4.2%)	19 (5.3%)	0.482
Psychiatric illness	14 (3.9%)	15 (4.2%)	0.850
Smoker	29 (8.1%)	20 (8.4%)	0.892
Substance abuse	18 (5.0%)	18 (5.0%)	1.00
Diabetes	1 (0.3%)	3 (0.8%)	0.316
Angiography, *n* (%)	18 (5.0%)	18 (5.0%)	1.000
Hypotensive on admission, *n* (%)	15 (4.2%)	16 (4.5%)	0.854
Received PRBC transfusion within 4 h, *n* (%)	60 (16.8%)	60 (16.8%)	1.000
ISS, median (IQR)	21 (13)	19 (13)	0.601
Injury, *n* (%)			
Kidney	102 (28.5%)	101 (28.2%)	0.934
Spleen	202 (56.4%)	187 (52.2%)	0.260
Liver	165 (46.1%)	176 (49.2%)	0.410
Pelvic fracture	115 (32.1%)	105 (29.3%)	0.418
Long bone fracture (humerus, femur, tibia/fibula)	126 (35.2%)	125 (34.9%)	0.938
Severe spinal injury*	2 (0.6%)	6 (1.7%)	0.155
AIS-Abdomen grade, *n* (%)			0.833
2	167 (46.6%)	172 (48.0%)	
3	111 (31.0%)	100 (27.9%)	
4	59 (16.5%)	63 (17.6%)	
5	21 (5.9%)	23 (6.4%)	

*VTE* venous thromboembolism, *eVTEP* early VTE chemoprophylaxis, *dVTEP* delayed VTE chemoprophylaxis, *ADHD* attention-deficit hyperactivity disorder, *PRBC* packed red blood cells, *ISS* injury severity score, *AIS* abbreviated injury scale

^*^Defined by AIS grade ≥ 3 for the spine

The matched eVTEP group demonstrated similar rates of VTE (0.6% vs. 1.7%, *p* = 0.16), DVT (0.3% vs. 1.1%, *p* = 0.18), and PE (0.3% vs. 0.8%, *p* = 0.32) compared to the dVTEP group. Among all 14-year-olds, only 1 patient experienced VTE. Likewise, only 1 patient in each of the 15- and 16-year-old groups had VTE. In contrast, among 17-year-olds, there were 5 patients with VTE. No deaths occurred in the eVTEP group, while 1 death was reported in the dVTEP group (*p* = 0.32) (Table 3). Of all included patients with VTE, the spleen was the most frequently injured organ (88.9%), followed by long bone fractures (66.7%), with a median ISS of 27 (Table 4).

**Table 3:** Outcomes for unmatched cohort and 1:1 propensity score-matched patients receiving early vs. delayed VTE chemoprophylaxis

Characteristic	eVTE	eVTE	*p*-value
*Unmatched cohort*			
Complications, *n* (%)			
VTE	2 (0.5%)	7 (1.2%)	0.255
Deep vein thrombosis	1 (0.2%)	4 (0.7%)	0.343
Pulmonary embolism	1 (0.2%)	4 (0.7%)	0.343
Mortality, *n* (%)	1 (0.2%)	1 (0.2%)	0.791
*Matched cohort*			
Complications, *n* (%)			
VTE	2 (0.6%)	6 (1.7%)	0.155
Deep vein thrombosis	1 (0.3%)	4 (1.1%)	0.178
Pulmonary embolism	1 (0.3%)	3 (0.8%)	0.316
Mortality, *n* (%)	0	1 (0.3%)	0.317

*VTE* venous thromboembolism, *eVTEP* early VTE chemoprophylaxis, *dVTEP* delayed VTE chemoprophylaxis, *LOS* length of stay, *ICU* intensive care unit, *NOM* non-operative management

**Table 4:** Demographics, injury patterns, and outcomes for adolescent patients with VTE

Characteristic	(*n* = 9)
Age, year, median (IQR)	17 (3)
Male, *n* (%)	6 (66.7%)
Comorbidities, *n* (%)	
Hypertension	0
ADHD	0
Psychiatric illness	1 (11.1%)
Smoker	2 (22.2%)
Substance abuse	0
Diabetes	0
Angiography, *n* (%)	0
Hypotensive on admission, *n* (%)	1 (11.1%)
Received PRBC transfusion within 4 h, *n* (%)	6 (66.7%)
ISS, median (IQR)	27 (38)
Injury, *n* (%)	
Kidney	4 (44.4%)
Spleen	8 (88.9%)
Liver	4 (44.4%)
Pelvic fracture	3 (33.3%)
Long bone fracture (humerus, femur, tibia/fibula)	6 (66.7%)
Severe spinal injury*	1 (11.1%)
Mortality, *n* (%)	0

*ADHD* attention-deficit hyperactivity disorder, *PRBC* packed red blood cells, *ISS* injury severity score, *AIS* abbreviated injury scale

^*^Defined by AIS grade ≥ 3 for the spine

In a subset analysis of 231 isolated splenic injuries, both VTE and NOM failure rates were similar (all *p* > 0.05). The ROC for VTE prophylaxis hours and VTE was 0.81, with VTEs occurring at 47, 176, and 289 h after the initiation of VTE chemoprophylaxis. Similarly, among 414 isolated liver injuries, VTE and NOM failure rates were comparable (all *p* > 0.05). The ROC for VTE prophylaxis hours and VTE was 0.41, with VTEs occurring at 13, 16, 75, and 89 h after chemoprophylaxis initiation. Lastly, in a subset analysis of 254 isolated kidney injuries, VTE and NOM failure rates showed no significant difference (all *p* > 0.05). The ROC for VTE prophylaxis hours and VTE was 0.50, with VTEs occurring at 13, 47, 75, and 101 h after chemoprophylaxis initiation. In all subgroup analyses, no difference in total hospital LOS was observed between patients receiving early versus delayed VTE chemoprophylaxis (all *p* > 0.05) (Table 5 and 6).

**Table 5:** Analyses for stratified isolated solid organ injuries

Characteristic	eVTEP	dVTEP	*p*-value
Spleen (*n* = 231)			
VTE rate	0	3 (2.1%)	0.168
NOM failure rate	1 (1.1%)	5 (3.5%)	0.265
Liver (*n* = 414)			
VTE rate	2 (1.4%)	2 (0.7%)	0.521
NOM failure rate	1 (0.7%)	8 (3.0%)	0.132
Kidney (*n* = 254)			
VTE rate	1 (1.1%)	3 (1.8%)	0.694
NOM failure rate	1 (1.1%)	4 (2.4%)	0.498

*eVTEP* early VTE chemoprophylaxis, *dVTEP* delayed VTE chemoprophylaxis, *NOM* non-operative management

Finally, we ran an additional analysis including the 61 patients that were excluded that died or were discharged within 48 h. We found the rate (eVTEP 2.0% vs. dVTEP 1.1%, *p* = 0.244) and adjusted risk (*p* = 0.779) of VTE were similar between both groups. Similarly, the rate (eVTEP 8.7% vs. dVTEP 5.1%, *p* = 0.254) and adjusted risk (*p* = 0.390) of failure of NOM were similar between both groups.

## Discussion

In this national study, we found that the spleen was the most common site of BSOI in adolescent trauma patients. Although the overall rate of VTE in adolescents with BSOI was extremely low (1%), more than 60% of VTE events occurred in 17-year-olds. Around 40% of patients undergoing NOM received early VTE chemoprophylaxis (first dose within 48 h); however, early VTE chemoprophylaxis did not result in a significant difference in VTE rates or failure of NOM when compared to similar adolescent patients who received delayed VTE chemoprophylaxis.

Timing of VTE chemoprophylaxis is of paramount importance for adult trauma patients. However, there is a paucity of data on this relationship in adolescent trauma patients who possess some similarities to adults and others to pediatric trauma patients. The comparable VTE rates between patients receiving early and delayed VTE chemoprophylaxis may be attributed to the rarity of VTE in adolescence, which more closely resembles pediatric trauma patients. Among hospitalized adolescents, those admitted after trauma are at a higher risk for VTE than non-injured patients due to post-trauma hypercoagulability, endothelial injury, and vascular stasis [21]. However, our study found that the overall VTE rate for adolescent patients with BSOI was approximately 1%, which is consistent with other single-center or multicenter studies on pediatric trauma populations (0.0001–2.1%) [5, 6]. In contrast, the incidence of VTE in adult trauma patients is 7–58% [7, 22]. With such a low incidence in adolescent trauma patients, it would be challenging to power a study that could demonstrate a statistically significant benefit.

The low VTE occurrence in pediatric and adolescent patients can be explained by several factors. First, there are two peaks in childhood VTE incidence: infants under 2 years old and adolescents [23]. In adolescents, the increased risk can be attributed to obesity, pregnancy, oral contraceptive use, and smoking. Hospitalized female adolescents are more likely to develop VTE than their male counterparts [24]. Younger children are less likely to have the aforementioned risk factors. Second, children and adolescents have significant physiological differences in the coagulation cascade compared to adults, with lower concentrations of procoagulants and nearly double the levels of thrombin inhibitors [25]. This is supported by the results of this study as the majority of VTE events (62.5%) occurred in the oldest adolescent patients (17-year-olds). Last, pediatric and adolescent patients have fewer systemic diseases, such as diabetes and hypertension, which can alter or damage the vascular endothelium [26]. These factors contribute to the rarity of VTE in adolescents.

Certain injury patterns pose a higher risk for VTE compared to others. Patients with pelvic fractures or spinal cord injury have an over tenfold higher risk of VTE compared to trauma patients without these injuries [27, 28]. Patients with BSOI are also at an increased risk of VTE, particularly if they receive interventional radiology intervention [29]. However, certain solid organs may be associated with a higher propensity to develop VTE in adolescent trauma patients compared to others. The spleen plays a critical role in the immunologic and hematologic systems and is responsible for disposing damaged red blood cells and platelets [30]. The injured spleen may be compromised in this regard, and release premature stored blood cells into circulation or poorly regulate the removal of damaged blood cells and platelets, thereby potentially contributing to clot formation [31]. In an analysis of nearly 30,000 trauma patients, those with splenic injury regardless of receiving a splenectomy or not had a nearly twofold higher risk of VTE [32]. These findings are corroborated by our study of adolescent trauma patients. The majority of VTE events we observed occurred in patients with a splenic injury. Furthermore, the only subgroup of isolated solid organ injury with a correlation between increased time to VTE chemoprophylaxis and VTE event was to the spleen. These findings suggest that splenic injury in particular poses the highest risk of VTE in adolescent blunt trauma patients.

Despite the relative rarity of VTE, early initiation of VTE chemoprophylaxis may be beneficial for adolescent trauma patients. Among experts in a national multidisciplinary study, there was consensus that hospitalized children under 12 years of age should not routinely be given VTE chemoprophylaxis. However, this same group of experts was unable to reach consensus on which age cutoff should begin receiving VTE chemoprophylaxis [15]. Since early VTE chemoprophylaxis does not increase the rate of failing NOM for adolescents with BSOI, early initiation of VTE chemoprophylaxis should be strongly considered in adolescent trauma patients with BSOI if no contraindication exists. This may be lifesaving for individual patients as VTE is a potentially preventable event, and in adult patients, the Joint Commission Taskforce has even identified VTE as a never-event. Similar efforts are now underway for pediatric and adolescent patients spearheaded by the Children’s Hospitals Solutions for Patient Safety [33].

Limitations of this study include those inherent to large database studies, such as selection and reporting bias. Additionally, the absolute difference in VTE rates between the two cohorts was 1.1%. To detect such a small difference, a sample size of 2948 patients (1474 in each group) would be needed. Therefore, our study was not sufficiently powered to detect a difference this small. Potential confounders in our study which are not included in the TQIP dataset include disruptions in administering VTE chemoprophylaxis (delayed or missed doses) and the almost certain variation of dosing of VTE chemoprophylaxis across trauma centers. Also, TQIP is confined to index hospitalization, thus likely underestimating the incidence of VTEs. Additionally, TQIP does not provide the temporal relation between a VTE event and chemoprophylaxis initiation. As such, we are unable to tell if the VTE event occurred prior to the initiation of VTE chemoprophylaxis. Another limitation is that TQIP only includes information regarding blood product transfusion for the first 4 h of hospital admission. Any subsequent blood product transfusion requirements are thus not captured in TQIP. Other factors related to VTE such as ambulation and use of sequential compression devices are also not available within TQIP. And finally, delayed angioembolization may be used as a marker for failure of NOM. However, TQIP only provides data regarding the use of angiography (with or without embolization) during the first 24 h, and only in patients requiring blood product transfusion within the first 4 h of arrival.

## Conclusions

The rate of VTE in adolescent patients with BSOI is exceedingly rare. However, more than 40% of adolescent patients with BSOI undergoing NOM receive early VTE chemoprophylaxis (first dose within 48 h). Importantly, early VTE chemoprophylaxis does not increase the rate of failure of NOM for adolescents with BSOI. The majority of VTE events occurred in older adolescents (17-year-olds) and in those with splenic injury. Unlike adult trauma patients, adolescent patients with BSOI receiving early VTE chemoprophylaxis had a similar rate of VTE and death, compared to adolescents receiving delayed VTE chemoprophylaxis. Future prospective research would be required to make a definitive statement about the safety of early VTE chemoprophylaxis in adolescent BSOI patients undergoing NOM.

## Supplementary Information

Below is the link to the electronic supplementary material.

Supplementary file1 (DOCX 33 KB)
